# P03-014 - Biological therapy for autoinflammatory disorders

**DOI:** 10.1186/1546-0096-11-S1-A212

**Published:** 2013-11-08

**Authors:** S Bujan Rivas, JI Arostegui Gorospe, F Martinez Valle, R Solans laque, JF Andres Cordon, J Ordi Ros, M Vilardell Tarres

**Affiliations:** 1 Internal Medicine, Hospital vall Hebron, Spain; 2 Immunology, Hospital Clinic i Provincial, Barcelona, Spain

## Introduction

Autoinflammatory diseases (AD) are innate immune system disorders, most of them with genetically identified basis. Usual therapeutic approach until last decade included non-steroidal antiinflamatories (NSAID), corticosteroids (CS) and colchicine. More recently, biological therapies (BT) have proved to be useful for refractory patients, especially those involving IL-1β blockade.

## Objectives

To describe and analyze the experience with BT of a single center cohort of adult patients diagnosed with autoinflammatory disorders, including familial Mediterranean fever (FMF), TNF-receptor antagonist periodic syndrome (TRAPS), Muckle-Wells syndrome (MWS), undefined periodic fever (UPF), Blau syndrome (BS) and periodic fever, aphtous stomatitis, pharyngitis and adenitis (PFAPA).

## Methods

Clinical files of 30 adult patients diagnosed with AD followed by the same clinical team of the Internal Medicine Service of Vall Hebron hospital were reviewed. Demographic, clinical, laboratory, and therapeutic data prior and after starting BT were collected. Data from BT treated patients included: reason for prescription, drug, dosage, treatment duration, response to treatment, tolerance and adverse effects related to the drug.

## Results

13 out of 30 patients (5 FMF, 1 TRAPS, 4 MWS, 1 UPF, 1 BS, 1 PFAPA) received BT: 7 patients (5 FMF, 1 BS, 1 TRAPS) received anti-TNF (6 etanercept 50 mg/weekly SC, 1 patient adalimumab 40 mg/15 d SC) and 10 patients (3 FMF, 4 MWS, 1 UPF, 1 BS, 1 PFAPA) received rIL-1RA anakinra 100 mg/d SC. 4 patients (2 FMF, 1 BS, 1 UPF) received first etanercept and were switched to anakinra due to incomplete / lack of response. Prescription reasons for BT were: first option 5/13 patients (etanercept for 1 TRAPS and anakinra for 4 MWS) and refractory disease in 8/13 patients (anti-TNF: 4 FMF, 1 UPF, 1 BS; anakinra: 3 FMF, 1 BS, 1 PFAPA, 1 UPF). Clinical improvement defined as reduction in number and/or intensity of attacks was achieved in 4/7 patients treated with anti-TNF and in 9/10 patients receiving anakinra. 6 patients, all treated with anakinra, presented adverse effects: 6 patients local erythema at punction site -2/6 were moderate reactions-, and 1 patient transient alopecia. No opportunistic infection was detected.

## Conclusion

Although BT are off-label indications in AD, its use has to be considered a helpful and safe therapeutic alternative for refractory cases of FMF, UPF, BS or PFAPA. In this serie, IL-1β blocking approach showed better response than anti-TNF with limited side effects.

## Competing interests

None Declared.

